# White Fibrous Papulosis of the Neck: A Case Report

**DOI:** 10.7759/cureus.25661

**Published:** 2022-06-04

**Authors:** Mariana Rios-Gomez, Jose A Ramos-Garibay, Martha E Perez-Santana, Mario A Rostro-Hernandez, Veronica Aguilera-Martinez

**Affiliations:** 1 Department of Internal Medicine, Hospital Regional de Pemex en Salamanca, Salamanca, MEX; 2 Department of Dermatology, Centro Dermatologico "Dr. Ladislao de la Pascua", Mexico City, MEX; 3 Department of Pathology, Hospital Regional de Pemex en Salamanca, Salamanca, MEX; 4 General Medicine, Private Practice, Salamanca, MEX; 5 Department of Dermatology, Hospital Regional de Pemex en Salamanca, Salamanca, MEX

**Keywords:** wfpn, rare skin disease, progressive disease, elderly onset, white fibrous papulosis of the neck

## Abstract

White fibrous papulosis of the neck is a rare entity, with a benign course and unknown pathogenesis. It is clinically characterized by the appearance of firm, persistent, usually asymptomatic, non-follicular papules located on the neck. We present the case of a 72-year-old patient who presented pruritic lesions on the neck whose biopsy was compatible with this entity.

## Introduction

White fibrous papulosis of the neck (WFPN) is a rare entity. The first case reported dates back to 1983 in Japan [1]. Since then, it has been described in other countries such as Italy, Great Britain, Saudi Arabia, France, Spain, Belgium, and China [2-3]. In Latin America, cases have been reported in Argentina, Brazil, and Chile [4-6]. In most cases, the nature of this pathology is asymptomatic, which causes its true incidence to be underestimated. We present a female patient with this rare disease.

## Case presentation

A 72-year-old female patient arrived at the Dermatology outpatient clinic due to pruritic “pimples” on her neck, which progressively increased in number over one year prior to the dermatology appointment. The patient had a medical history of type 2 diabetes mellitus, systemic arterial hypertension, end-stage kidney disease on maintenance hemodialysis, hypothyroidism, and hypersensitivity to trimethoprim-sulfamethoxazole.

**Figure 1:**
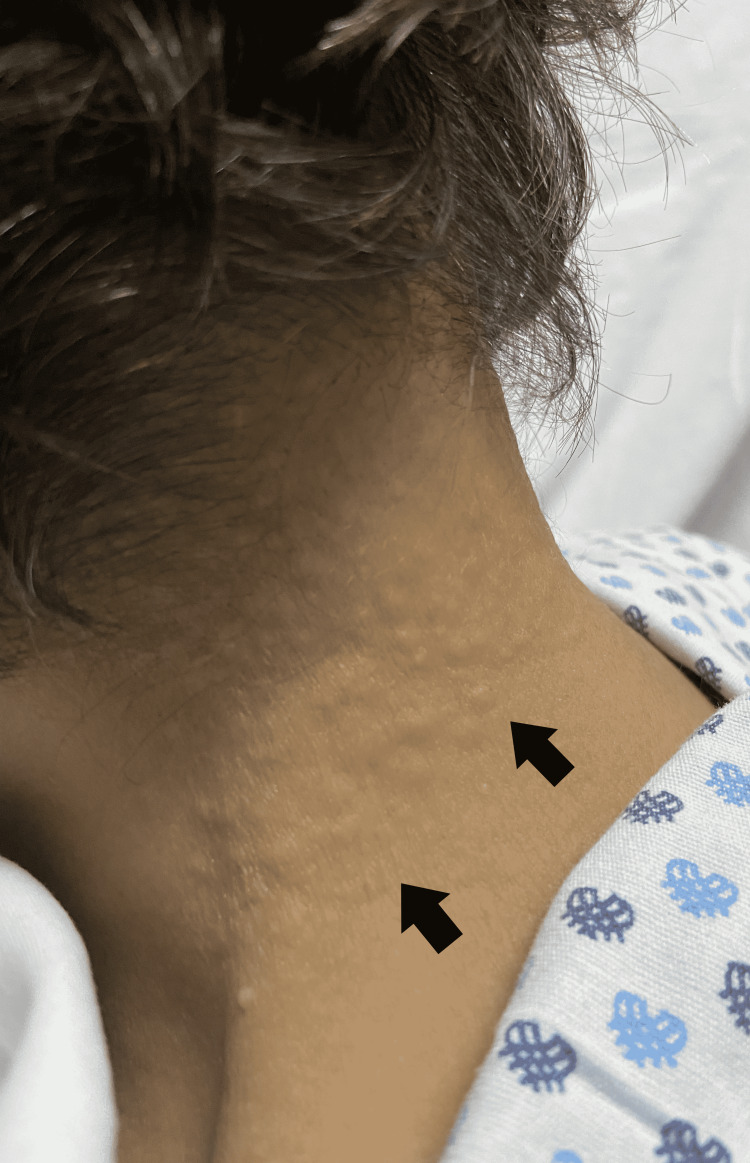
Papulous eruptions measuring 2-3 mm located on the side of the neck (black arrows)

**Figure 2:**
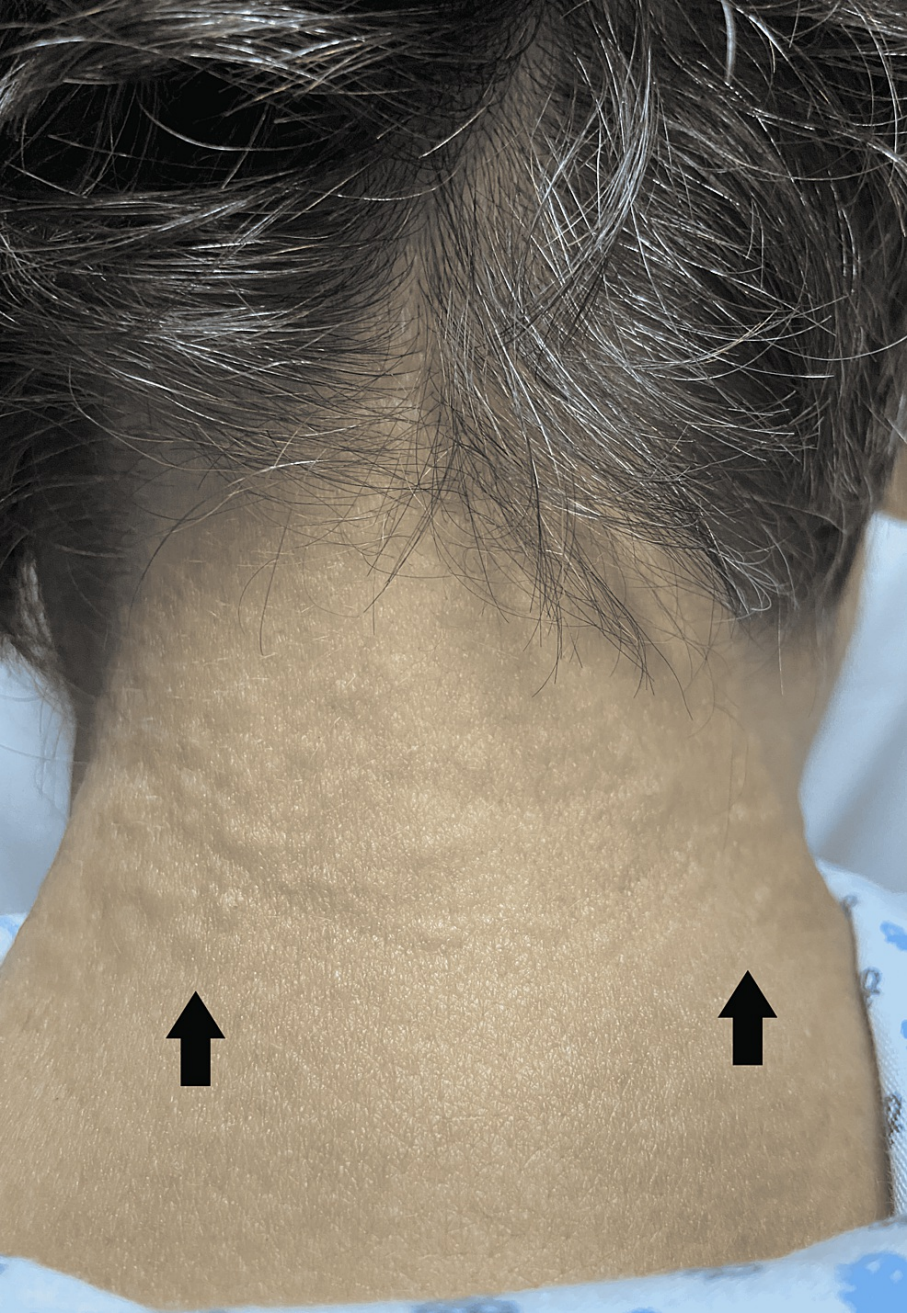
Papulous eruptions measuring 2-3 mm located on the posterior part of the neck (black arrows)

Physical examination showed a skin disease localized on the posterior and lateral sides of the neck (Figure 1) consisting of multiple papular lesions measuring 2 mm in diameter, skin-colored, and hard to touch (Figure 2).

**Figure 3:**
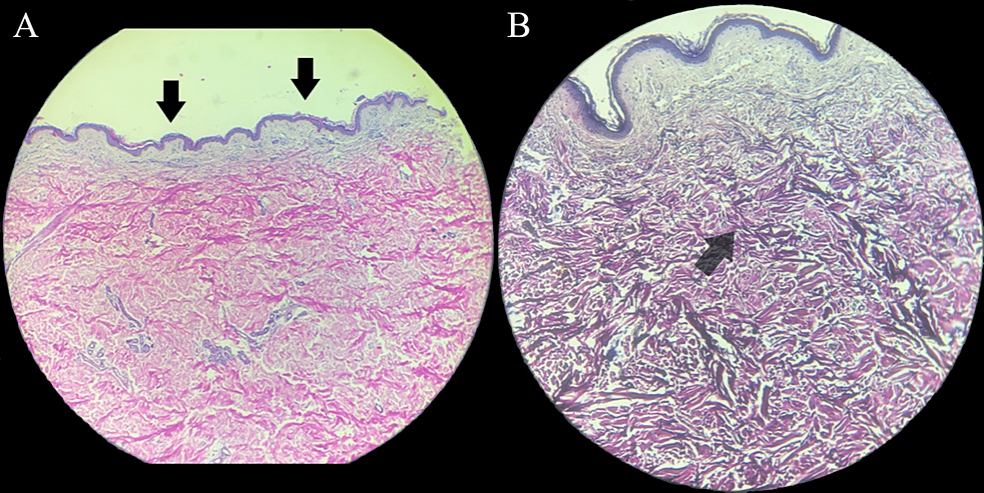
A: Histological examination with hematoxylin & eosin staining (10X) revealed a folded epidermis with hyperkeratosis and orthokeratosis with thickening of collagen fibers in the superficial and medium dermis (black arrows). B: Argentic staining for elastic fibers (10X) revealed a decrease in elastic fibers, with fragmentation in some of them (transparent arrow)

An incisional biopsy from the posterior region of the neck was taken, which showed a slightly folded epidermis with orthokeratosis, and atrophy of the spiny layer but conserving most of its interpapillary processes; thickening of the collagen fibers was observed in the superficial and medium dermis, with no change in its orientation (Figure 3A). Argentic staining identified a decrease in the elastic fibers and fragmentation (Figure 3B). Thus, the diagnosis of WFPN was made [7]. The patient did not request cosmetic treatment and remains stable at one year of follow-up.

## Discussion

WFPN was first reported in Japan in 1983 by Shimizu et al. who reviewed 32 patients with asymptomatic papules on the neck. The histological examination of these patients showed fibrosis in the papillary dermis and, in some cases, slight changes in the elastic fibers [1].

In 1992, Riongioletti and Rebora reported two elderly women with similar neck lesions, which they described as “acquired elastolysis of the papillary dermis simulating elastic pseudoxanthoma.” The histological examination revealed papillary elasticity without fibrosis. In 1997, Balus et al. considered there was an overlap between the clinical and pathological characteristics of these entities, and the term “fibroelastolytic papulosis of the neck” was proposed [8].

White fibrous papulosis appears as monomorphic papulose eruptions about 2 to 3 millimeters in diameter, which can be isolated or confluent. These papulose eruptions are firm, non-follicular, and with a smooth surface. The lesions can be round or oval, of normal skin color, ivory white or yellow, and they are asymptomatic and sometimes pruritic [1]. They are mainly located around the neck but can also affect the upper part of the trunk, abdomen, armpits, and limb folds [1,3]. Most of the patients are women over 60 years of age, but there are case reports of younger ages [1,3], the youngest being 26 years old [1]. Lesions appear progressively and do not go away over time.

The pathogenesis of this disease is unknown. Some authors believe that sun exposure is involved, by accelerating the skin’s aging process [1]. Other authors think it is related to chrono-aging, which is why they consider this papulosis a “fibroelastolytic pattern of intrinsic skin aging” [9].

There are no reports of association with drugs, diseases, family history of similar lesions, or racial predilection to date. However, Catacora et al. reported a case of papillary dermal elastolysis type pseudoxanthoma elastic with the involvement of hormonal factors in young women [10].

Histological findings by hematoxylin and eosin stain include superficial dermal fibrosis with little or no change in elastic fibers, as well as a variable thickening of collagen fibers in the superficial dermis. With the Verhoeff-Van Gieson stain, specific for elastic fibers, the content of these fibers is shown to be slightly reduced or absent. In transmission electron microscopy, no significant morphological changes are shown [2-3,5]. Dermatoscopy reveals white, homogeneous, circumscribed areas with thin dotted or short vessels, without follicular involvement [11].

The differential diagnosis must be established with other papular eruptions that appear on the neck. It should first include elastic pseudoxanthoma, which presents ocular and cardiovascular manifestations, with histology showing the absence of fragmentation of elastic fibers and calcium deposits. Acrocordons, fibrofolliculomas, and trichodiscomas in Birt Hogg-Dube syndrome, Buschke-Ollendorf syndrome, cutis rhomboidalis nuchae, eruptive xanthomas, and perifollicular elastosis should also be considered (Table 1) [4,12].

**Table 1:** Differential diagnosis of white fibrous papulosis of the neck TGF, transforming growth factor; Er: YAG laser, Erbium-doped Yttrium aluminum garnet laser Source: [13-17]

	DEFINITION	EPIDEMIOLOGY	PHYSIOPATHOLOGY	CLINICAL FEATURES	DIAGNOSIS	TREATMENT	PROGNOSIS
ELASTIC PSEUDOXANTHOMA	A multisystem disorder that primarily affects the skin, eyes, and cardiovascular system characterized by progressive and degenerative calcification of the elastic fibers.	1 in 25,000-100,000. Female predominance 2:1.	ABCC6 gene defect on chromosome 16p13.1 with an autosomal recessive inheritance.	Skin signs: yellow cobblestone lesions on areas of bending. Ophthalmological manifestations: angioid stretch marks, peau d’orange, maculopathy. Cardiological manifestations: intermittent claudication, coronary artery disease, arterial hypertension, angina, myocardial infarction, congestive heart failure, restrictive cardiomyopathy, and valvulopathies.	Histology: calcium deposit with injured, swollen, clustered, and fragmented elastic fibers between the reticular and deep dermis. Electron microscopy: Mineralization of the elastic fibers starts at the core, and as calcification progresses fragmentation of the fibers occurs.	Ophthalmological and cardiovascular evaluation. Laser photocoagulation to prevent retinal bleeding, adequate cardiovascular risk factors management, moderate dietary calcium intake, and antiplatelet/ anticoagulants avoidance. Genetic counseling. Plastic surgery.	Usually normal life expectancy. Morbidity and mortality depend on the systemic extent of the disease. Pathological changes are irreversible.
ACROCHORDONS (SKIN TAGS)	Pedunculated benign cutaneous neoplasia.	Present in 50% of the adults. More frequent with increasing age, during the second trimester of pregnancy, in obese patients, in diabetic patients, and Birth-Hogg-Dube syndrome.	Associated to increased risk of diabetes mellitus and hypertension.	Lesions are located in sites of friction, axillary, cervical, inframammary, and inguinal regions. Asymptomatic until there is a trauma to the lesions. They appear as a red or black lesion when there is torsion of their peduncle.	Histology: Lesions consist mainly of fibers of collagen, fat, and other types of tissues such as blood, blood vessels, mast cells, Langerhans cells, and dermis.	Aesthetic removal with fine scissors, cryosurgery with liquid nitrogen, and electrodesiccation.	Lesions rarely reappear after removal. New lesions may develop in areas of predisposed skin.
FIBROFOLLICULOMAS AND TRICHODISCOMAS	Annexal tumors that arise from or around the hair follicles.	Depends on the underlying pathology.	Unknown. Associated with Birth-Hogg-Dube syndrome.	Papules of 2-4 mm in the shape of a meat-colored dome. They appear on the face (chin, nose, cheeks, ears, and eyebrows). Both lesions are asymptomatic and indistinguishable from each other on visual examination.	Histology fibrofolliculoma: Dilated central follicular infundibulum, epithelial strands of basaloid cells emanating from the infundibulum of the hair follicle. Histology trichodiscoma: Proliferation of connective tissue and fibrous stroma, located near a hair follicle.	Surgical excision and combined CO2/Er: YAG laser.	Depends on the underlying pathology.
BUSCHKE OLLENDORFF SYNDROME	Genodermatosis with connective tissue nevus, osteopoikilosis, and an autosomal dominant inheritance.	It affects both sexes equally. Incidence of 1 in 20,000. Connective tissue nevus appears before puberty,	Mutations that cause a loss of function in the LEMD3 gene on chromosome 12q14, inhibit the function of the TGF, which induces the production of extracellular matrix components like type I collagen, elastin, and fibronectin.	Linear cortical hyperostosis is seen in long bones in an x-ray. Connective tissue nevus is seen as papules, plaques, and yellowing or skin-colored nodules on thighs and buttocks.	Histology of connective tissue nevus: Increased elastic fibers or collagen fibers.	No specific treatment is required.	Benign and usually asymptomatic disease (25% of patients experience bone pain and joint edema).
CUTIS ROMBOIDALIS NUCHAE	Manifestation of prolonged sun exposure and resulting skin damage that occurs at the back of the neck.	Elderly with fair skin who were exposed to ultraviolet radiation.	Sun-induced skin disorder in which ultraviolet radiation causes the degeneration of elastin and collagen fibers.	Diffuse thickening on the back of the neck with yellowing of the skin, and formation of deep grooves which results in a typical irregular rhomboidal pattern.	Histology: Thickening of the epidermis and abnormalities in the composition of the dermis.	Photoprotection, topical retinoids, and fluorouracil.	Association with actinic keratosis and basal cell carcinoma.

There is no established treatment for injury remission. Although the lesions are benign, patients may appreciate these lesions as cosmetically unpleasant, so treatment should focus on flattening the lesions to achieve an aesthetic result acceptable to patients.

The use of 1550-nm fractional non-ablative laser, Erbium-doped Yttrium aluminum garnet laser, and carbon dioxide (CO_2_) laser have been reported. Other treatment options include photoprotection and topical preparations with retinoids [5,18] or antioxidants that reduce age-induced free radicals [19]. Tacrolimus 0.1% twice daily has demonstrated an improvement in pruritus [20].

## Conclusions

WFPN is a rare entity, with a characteristic clinical picture and unknown pathogenesis. There are few cases reported to date, so full documentation and publication are of paramount importance. These will help us know the true prevalence of WFPN, and the factors associated with it, thus resulting in a better characterization of this rare disease. Also, it's important to highlight that these lesions tend to be pruritic and aesthetically unacceptable, they affect the quality of life of those who suffer from it, so it is necessary to implement new treatment strategies that lead to the well-being of our patients. We hope this case report will call the attention of other health professionals and improve the detection of WFPN.
